# Perceived unmet needs and impact on quality of life of patients living with advanced bladder cancer and their caregivers: results of a social media listening study conducted in five European countries

**DOI:** 10.1186/s12885-024-13092-x

**Published:** 2024-11-25

**Authors:** Stephane Schuck, Paul Loussikian, Adel Mebarki, Joelle Malaab, Pierre Foulquié, Manissa Talmatkadi, Mairead Kearney

**Affiliations:** 1 Kap Code, 146 Rue Montmartre, Paris, 75002 France; 2 Merck Healthcare KGaA, Darmstadt, Germany

**Keywords:** Advanced bladder cancer, Europe, Real-world, Retrospective, Quality of life, Social media

## Abstract

**Background:**

Advanced bladder cancer (aBC) is a significant health concern in Europe and has a poor prognosis. Patients with aBC face numerous unmet needs and challenges that significantly impact their quality of life (QoL). This study aims to analyze social media data from five European countries to address gaps in our understanding of the unmet needs, challenges, and impact on QoL in European patients with aBC and their caregivers.

**Methods:**

This retrospective, real-world study includes public social media posts geolocated in France, Italy, Germany, Spain, and the UK, posted between October 2017 and January 2022. To enhance the filtering process, natural language processing methods and specific algorithms were used to remove irrelevant content and retain posts from patients and caregivers. QoL impacts were identified using a deep-learning algorithm, followed by qualitative analysis. Unmet needs were analyzed via annotation of messages and the saturation method.

**Results:**

A total of 1670 posts from 1396 users (699 posts from 546 patients and 971 posts from 850 caregivers) discussing aBC in 91 publicly available online sources were identified. Half of patients were male (*n* = 272, 49.8%) while more caregivers were female (*n* = 474, 55.8%), with an average age of 58.2 and 35.2 years, respectively. Patients and caregivers expressed an impact on QoL, wherein 40.0% (558/1396) of users mentioned at least an impact on one aspect of QoL. Among those, 56.8% (317/558) and 48.6% (271/558) of users expressed physical and psychological challenges, respectively. Most unmet needs identified belonged to two main categories: transversal, i.e., arising throughout the patient’s care pathway (307/1092 [28.1%]), and disease specific (295/1092 [27.0%]). Main challenges included worsening of the disease (*n* = 141, 12.9%), psychological impact (*n* = 112, 10.3%), and need to share experiences and seek support (*n* = 94, 8.6%).

**Conclusions:**

This social media listening study demonstrated the profound emotional and physical burden on patients with aBC and their caregivers, and a genuine need for support and an outlet to discuss their challenges, particularly in terms of managing the illness. These results underscore the importance of enhancing education for both patients and caregivers and the necessity for more effective systemic cancer therapies and better palliative care alternatives.

**Supplementary Information:**

The online version contains supplementary material available at 10.1186/s12885-024-13092-x.

## Background

Bladder cancer (BC) is a significant health concern in Europe, with a considerable impact on patients and healthcare systems. In Europe, BC is the fourth most common cancer among men and the ninth most common among women [1, 2], with the same ranking among men in France, Germany, Italy, and Spain; BC is the sixth most common cancer in the United Kingdom (UK) [3]. The overall economic burden of BC in the European Union (EU) was estimated at several billion euros, with healthcare expenditures accounting for >50% of this total [4].

Advanced BC (aBC), which refers to disease that has spread beyond the bladder to nearby tissues (locally advanced) or distant organs (metastatic), poses unique challenges in terms of management and prognosis. It is associated with poorer outcomes and a higher risk of metastasis, leading to increased morbidity and mortality [5].

Patients with aBC face numerous unmet needs and challenges that significantly impact their quality of life (QoL) [6–8]. The aggressive nature of the disease and the complex treatment regimens, including surgical interventions, chemotherapy, radiation therapy, and immunotherapy, often result in physical, emotional, and psychosocial distress. Patients may experience symptoms such as pain, fatigue, urinary dysfunction, and decreased mobility, leading to a significant decline in their overall well-being [9, 10].

In addition, the uncertainty surrounding the disease’s course, the impact on daily activities and the fear of disease progression and recurrence can contribute to increased anxiety and depression and decreased QoL [11, 12]. Caregivers who play a crucial role in providing support and assistance to patients, often experience emotional strain, caregiver burden, and decreased well-being [9].

Despite the recognition of these unmet needs and challenges, significant gaps still exist in our understanding of the specific experiences and needs of patients with aBC and their caregivers in Europe. It is crucial to explore these topics in depth to develop comprehensive interventions and support strategies that address the needs of patients and caregivers.

While clinical studies have led to the development of new treatment options and better outcomes in the management of aBC [1, 13–16], understanding the experiences and perceived challenges of patients and caregivers is essential to designing comprehensive care programs. Traditional research methods have limitations in capturing the nuances of patients’ and caregivers’ experiences. However, the rise of social media platforms has provided a unique opportunity to gather real-time data and gain insights into the challenges faced by individuals living with aBC and their caregivers [17–19]. These platforms have emerged as valuable resources for generating insights and addressing critical gaps in our understanding of aBC. By leveraging the power of social media data, we can discover perspectives on patient experiences, treatment decision-making, information-seeking behaviors, and the psychosocial impact of advanced disease [20]. Analyzing social media discussions and experiences shared by patients and caregivers provides valuable insights into their perspectives, concerns, and unmet needs [9]. These insights can help guide the development of patient-centered interventions, supportive care programs, and healthcare policies that improve the overall well-being and outcomes in patients and caregivers affected by aBC.

 This study aimed to address the gaps in our understanding of the unmet needs, challenges, and impact on QoL of patients with aBC and their caregivers through the analysis of social media data from five European countries.

## Methods

### Study design and population

This observational, retrospective, real-world study included data retrieved from social media posts of patients with aBC and their caregivers. The messages, written in the respective languages of the countries, were geolocated in five European countries (the Eu5): the UK, France, Italy, Spain, and Germany. The duration of the study spanned from January 2017 to October 2022.

The data collected consisted of messages sourced from public platforms such as X (formerly Twitter) and health-related forums such as Doctissimo and Macmillan.org. Due to restricted data access, only public Facebook pages and open groups were analyzed; Instagram and WhatsApp were excluded from this study. To gather relevant data, a list of keywords associated with BC (e.g., bladder, urothelial, and ureter combined with cancer, tumor, and carcinoma) was meticulously compiled with the assistance of linguists, covering each country’s respective language. Secondary filters were then applied to focus specifically on the advanced stage of the disease (e.g., metastatic, end stage, 3a, 3b, 4a, 4b, and invasive). To account for potential misspellings and maximize exhaustivity, multiple spellings of keywords were considered. The complete list of these carefully selected keywords was subsequently used in the extraction query (Supplementary Material).

Data extraction was performed by the Brandwatch^®^ extractor (Cision Ltd.) [21]. Initially, Brandwatch^®^ extractor scanned publicly available sources and gathered posts that included keywords matching the ones in the query. Concurrently, a web crawling or data collection process collected information from publicly accessible Facebook pages. Posts were retrieved along with their associated metadata (e.g., author and publication date). As a result, the dataset was created in Microsoft Excel.

No distinction was made in the treatment of posts obtained from different platforms, ensuring equal consideration and analysis across all sources. During the pre-processing phase, pertinent messages were selected based on exclusion criteria. Posts consisting of five words or less, as well as those exceeding 10,000 characters, were disregarded due to their lack of relevance. Additionally, duplicates were removed as were sources deemed unreliable or irrelevant to our study (e.g., advertising websites, and forums about cars, pets, or animals). Lastly, posts not written in English, French, German, Spanish, or Italian were excluded.

To enhance the filtering process, natural language processing methods and specific algorithms (Kap Code proprietary information) were used to filter out irrelevant content and identify posts from patients and caregivers. The algorithm had previously been developed using a training set of 12,330 messages across different health domains, including dermatology, tobacco use, and oncology. Our methodology involved a sequential pipeline consisting of two XGBoost classifiers [22]: one specifically designed to identify caregivers’ experiences, and the other to detect patients’ experiences. This approach enabled us to accurately categorize each post as belonging to a patient, a caregiver, or neither. The classifiers are constructed based on features that combine pronouns and lexical fields describing relatives and medical conditions (e.g., “my [pronoun] father [relative] has cancer [medical condition]”). To train the algorithm, we initially identified the caregivers using the complete dataset. Subsequently, we applied the algorithm again to the remaining dataset, excluding the previously identified caregiver messages, to determine patients’ messages. The performance evaluation yielded F1 scores [23], which measure accuracy by combining precision and recall, of 88% for the caregiver classifier and 87% for the patient classifier. The analysis focused solely on examining posts made by patients and caregivers in the current research. This algorithm has been used in previous work [24–26].

### Data analysis

#### Demographics

We ascertained patient or caregiver age and sex through a manual examination of the text, particularly when this information was explicitly provided, as exemplified below:

*“[…] my father passed away 2 months ago at the age of 71 due to metastatic bladder cancer”.*

In cases where age and sex information were not explicitly mentioned, we categorized the data as “undetermined.”

#### Impact on QoL

To comprehensively assess the impact on QoL, we developed a deep learning algorithm [27]. This algorithm was designed to identify relevant content by employing regular expressions (Regex) to detect specific keywords and phrases indicative of the different possible impacts. The algorithm categorized messages into five dimensions to gauge the impact on QoL: (1) the physical impact, with messages featuring keywords related to physical health concerns, symptoms, or limitations; (2) the psychological impact, with messages expressing emotional distress, mental health challenges, or psychological well-being; (3) the social impact, with posts highlighting changes in social interactions, support systems, or isolation due to health issues; (4) the financial impact, with messages discussing financial difficulties or burden caused by healthcare expenses or work limitations; and (5) the impact on daily activities, with posts revealing alterations in daily routines, activities, or lifestyle due to health-related factors.

The algorithm was refined and validated based on established QoL questionnaires, the EQ-5D [28], and the SF-36 [29] to ensure accuracy and consistency in the categorization process. Subsequently, two independent evaluators (Paul Loussikian and Joelle Malaab) performed a qualitative analysis by annotating messages to validate and interpret our findings.

#### Unmet needs and challenges

A deep learning algorithm was applied to identify main categories of challenges. Unmet needs of patients with aBC and their caregivers were identified via qualitative analysis conducted by the two independent evaluators. We annotated messages using an extensive grid comprising eight main categories, encompassing 54 distinct challenges and unmet needs faced by patients and caregivers. Based on the content of the message, evaluators categorized the message in one or more of the main categories featured in the grid. Evaluators also noted whether the message pertained to an unmet need of a patient, caregiver, or both. This algorithm has been used in previous work [24, 30].

Because of the wide range of unmet needs, we used data saturation to acquire a comprehensive sample of expressed challenges. Since there are no established guidelines for determining saturation in the context of social media content, we opted to use this innovative approach which has attained widespread acceptance as a methodological principle in qualitative research [31–33]. The saturation method involves collecting data to a point where further data collection is unnecessary. This technique was applied to our dataset by successively analyzing samples of messages until no further insights were found. We qualitatively analyzed random samples taken from all the available posts, with each sample being deliberately chosen to represent 5% of the total size; this process continued until saturation was reached. Achieving saturation was marked by the point where two consecutive samples failed to introduce more than two newly identified unmet need categories. After saturation was reached, two additional samples were qualitatively analyzed to ensure the validity of our findings.

#### Statistical analysis

For the anonymized content, only aggregated qualitative findings are reported. Manual curation was used to analyze and map, in detail, challenges and unmet needs expressed by users. All data were analyzed using descriptive statistics. Categorical data were described using the number of posts and/or percentages.

## Results

### Description of the population and posts

**Fig. 1:**
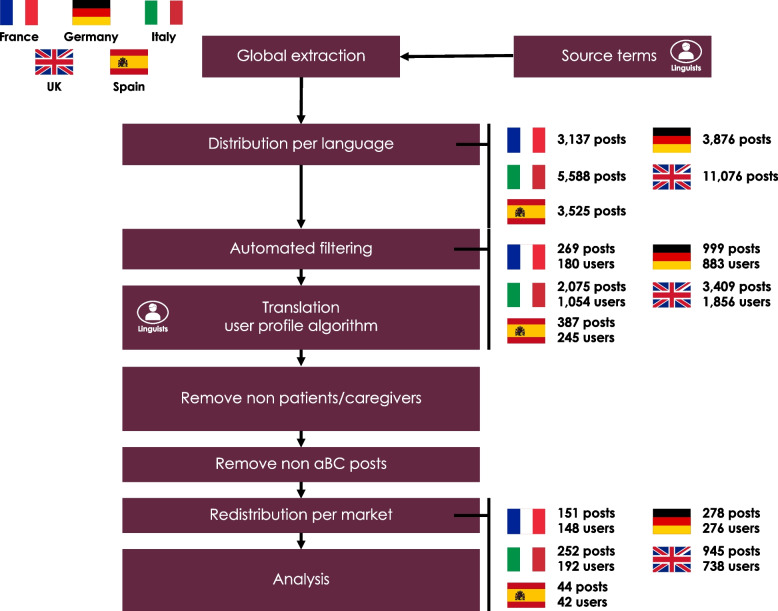
Extraction process. aBC, advanced bladder cancer

The extraction process produced a total of 27,202 messages from the Eu5 combined. After data cleaning, 1670 messages from 1396 users were retained (699 posts by 546 patients and 971 posts by 850 caregivers) (Fig. 1).

The UK had the highest number of posts (945 [56.6%]), followed by Germany (278 [16.6%]), Italy (252 [15.1%]), France (151 [9.0%]), and Spain (44 [2.6%]) (Table 1).

**Table 1:** Geographic distribution of posts and users

Country	Total posts, *n* (%)	Total users, *n* (%)	Patient posts, *n* (%)	Patients, *n* (%)	Caregiver posts, *n* (%)	Caregivers, *n* (%)
**UK**	945 (56.6)	738 (52.9)	419 (59.9)	291 (53.3)	526 (54.2)	447 (52.6)
**Germany**	278 (16.7)	276 (19.8)	150 (21.5)	150 (27.5)	128 (13.2)	126 (14.8)
**Italy**	252 (15.1)	192 (13.7)	82 (11.7)	57 (10.4)	170 (17.5)	135 (15.9)
**France**	151 (9.0)	148 (10.6)	40 (5.7)	40 (7.3)	111 (11.4)	108 (12.7)
**Spain**	44 (2.6)	42 (3.0)	8 (1.2)	8 (1.5)	36 (3.7)	34 (4.0)
**Total**	**1670 (100)**	**1396 (100)**	**699 (100)**	**546 (100)**	**971 (100)**	**850 (100)**

Messages were retrieved from 91 available public sources; some were global sources such as X (formerly Twitter; 261 [15.6%]) and Facebook (14 [0.8%]), but the majority were obtained from country-specific health forums. The UK’s Macmillan Cancer Support had the greatest number of posts (390 [23.4%]), followed by other health-related forums such as Germany’s Blasenkrebs Online-Selbsthilfegruppe (202 [12.1%]) and the UK’s HealthUnlocked (170 [10.2%]) (Table 2).

**Table 2:** Top 10 data sources

Forum/social media	Country	Posts, *n* (%)
Macmillan Cancer Support	UK	390 (23.4)
X (formerly Twitter)	Global	261 (15.6)
Blasenkrebs Online-Selbsthilfegruppe	Germany	202 (12.1)
HealthUnlocked	UK	170 (10.2)
Cancer Research UK	UK	156 (9.3)
Doctissimo	France	94 (5.6)
Aimac	Italy	89 (5.3)
Medicitalia	Italy	44 (2.6)
Ligue contre le cancer	France	30 (1.8)
Facebook	Global	14 (0.8)
Others	Global	220 (13.2)

Among the patient posts, approximately half were written by males (272 [49.8%]) with an average age of 58.2 years. In contrast, caregiver posts were primarily written by females (474 [55.8%]) with an average age of 35.2 years. Additionally, most individuals referred to in caregiver posts were male patients (577 [67.9%]) with an average age of approximately 67.7 years (Table 3).

**Table 3:** Patient and caregiver characteristics

Characteristics	Patients	Caregivers
**No. of users**	546	850
**No. of posts**	699	971
**Users’ sex**,** n (%)**		
Female	158 (28.9)	474 (55.8)
Male	272 (49.8)	198 (23.3)
Undetermined	116 (21.3)	178 (20.9)
**User age**,** n (%)**		
<40 years	19 (3.5)	39 (4.6)
40–59 years	76 (13.9)	47 (5.5)
≥60 years	58 (10.6)	79 (9.3)
Undetermined	393 (72.0)	685 (80.6)

### Impact on QoL

**Fig. 2:**
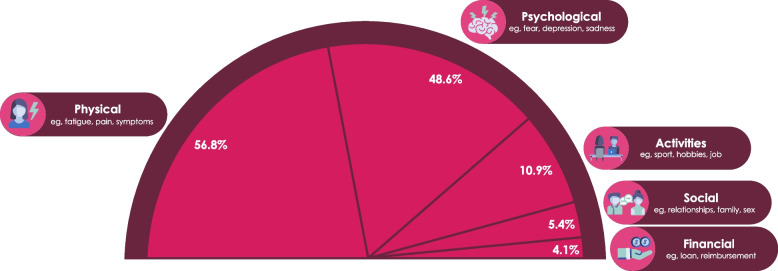
Proportion of internet users expressing an impact on quality of life

The analysis identified that 40.0% of users (558/1396) mentioned a negative impact on one aspect of QoL. Among the 558 users, 56.8% (317/558) expressed a physical impact, 48.6% (271/558) described a psychological impact, 10.9% (61/558) expressed an impact on their daily activities, 5.4% (30/558) expressed an impact on their social life, and 4.1% (23/558) expressed a financial impact (Fig. 2). It is worth noting that a message may have contained several aspects of QoL.

**Fig. 3:**
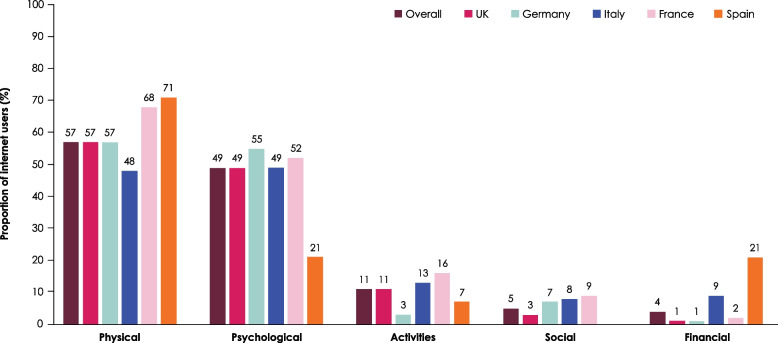
Proportion of internet users expressing an impact on quality of life by dimension and country

The physical and psychological impact on QoL was reported in the majority of cases (Fig. 3). Notably, a negative impact on physical well-being was reported consistently across all countries, with 56.8% of users reporting physical challenges. Patients and caregivers in the UK and Germany reported a comparable impact on physical aspects, with approximately 57.0% of users in both countries (131/230 in the UK; 40/70 in Germany) experiencing such challenges. In contrast, users reported higher physical impact rates in France (60/88 [68.2%]) and Spain (17/24 [70.8%]). Psychological well-being was also affected, with 49% of overall patients and caregivers experiencing psychological challenges. Users in Germany reported experiencing the highest psychological impact (39/71 [54.9%]), while those in Spain reported the lowest psychological impact (5/24 [20.8%]). Activities and social well-being were less commonly impacted, with only 10.9% and 5.4% of users, respectively, reporting challenges in these dimensions. Users in Germany reported the lowest percentage of impact on activities (2/71 [2.8%]). Finally, financial challenges were reported by a minority of patients and caregivers (3.9% overall). Users in Spain were the exception as they reported a higher financial impact than users in the other countries (5/24 [20.8%]). The lowest financial impact was reported by users in the UK (2/230 [0.9%]) and Germany (1/71 [1.4%]) (Fig. 3).

Users who expressed a physical impact mainly described pre-diagnosis symptoms, including blood in the urine, frequent visits to the bathroom, and severe pain. Users also reported an altered general state of being and sequelae related to surgery and procedures that the patients had undergone, as illustrated in a caregiver’s message below:

*“Hello*,* my mom has been diagnosed with advanced bladder cancer. She probably has metastasis on the bones. She hasn’t eaten or drunk much for several months*,* is very tired*,* has a lot of pain and can’t walk for long” (UK).*

The psychological impact included reports of poor mental health for caregivers. They described the mental distress of accompanying their sick relatives throughout their healthcare journey. The fear of losing their loved one and the feeling of helplessness were commonly reported in the caregivers’ posts. The patients expressed a feeling of desperation, especially if they had received a confirmed diagnosis. This was coupled with feelings of loneliness, being overwhelmed, and confusion. Furthermore, users voiced their concern about the future, mainly the potential worsening of their condition, and anxiety preceding medical examinations and check-ups, as described in the following message:

*“Hello everyone! I want to share my story with you: I am a 32-year-old girl. Just 5 years ago*,* I was diagnosed with a bladder sarcoma*,* which is a very serious tumor*,* especially for a woman. […] I dread the check-up scans: every time I have this one*,* which is every six months*,* I feel as if my life has stopped. At least for now*,* I can say I did. A hug to everyone and good luck” (Italy).*

Furthermore, aBC was described to impact users’ daily activities, manifesting as frequent urination, sometimes leading to embarrassing situations. With time, patients with aBC reported a loss of autonomy, as some of them who are greatly limited by the disease find themselves dependent on their caregiver for most of their activities. They reported this change as radical and hard to accept, especially for patients who were previously fit and athletic. Furthermore, users reported disturbed essential activities such as eating, drinking, and sleeping, which forced patients to adapt their lifestyle to manage their situation and to avoid impacting their relatives or caregivers’ lives as well, as shown in the following message:

*“I had all my tests done in April and was found to have invasive bladder cancer. It has made my bladder so bad that I am now incontinent and have to wear a bag all the time. I continue to have urinary tract infections and the pain is now so bad that I sleep downstairs on a chair so as not to disturb my wife’s sleep” (Germany).*

The impact on social aspects revolved around a feeling of isolation. With family and friends far away, depression or an unwillingness to inflict pain on their relatives, patients may find themselves voluntarily or involuntarily isolated when dealing with the disease. This isolation could sometimes have affected the caregiver as well. This ultimately affected relational aspects of patients’ lives, as expressed in the message below:

*“Hello everyone*,* once again thank you for these messages. It makes me feel really good to know that there are people around me to give me a little support*,* since many of my so-called friends don’t dare to come and see me or call me anymore*,* because they don’t know what to say!” (France).*

Finally, the financial impact of the disease was described as difficulty in accessing social assistance and benefits according to some patients who were no longer able to work. This also impacted their caregivers. The private healthcare sector, although recognized for the quality of its care, was criticized for the fees charged for treatment. This is described in the following message:

*“If you can pay them. [NAME] saved my partner eight years ago from bladder cancer in the Social Security. An excellent doctor but like others he will settle only in the private sector” (Spain).*

### Unmet needs

#### Main categories identified

The utilization of both the categorization algorithm and manual annotation in tandem facilitated the identification of 1092 instances of challenges and unmet needs across all posts, with 382 occurring in patient posts and 710 in caregiver posts. Notably, no noteworthy variations were observed in the challenges experienced by patients and their caregivers across the Eu5 countries.

Most unmet needs and challenges identified belonged to two main categories: transversal, i.e., arising throughout the patient’s care pathway (307 [28.1%]), and disease specific (295 [27.0%]). All results by category are shown in Table 4.

**Table 4:** Categories of challenges and unmet needs faced by patients and caregivers

Category	Occurrence in all posts combined, *n* (%)	Occurrence in patient posts, *n* (%)	Occurrence in caregiver posts, *n* (%)
**Transversal**	307 (28.1)	120 (31.4)	187 (26.3)
**Disease specific**	295 (27.0)	88 (23.0)	207 (29.2)
**Treatment specific**	141 (12.9)	49 (12.8)	92 (13.0)
**Care and follow-up specific**	117 (10.7)	50 (13.1)	67 (9.4)
**Patient-environment specific**	78 (7.2)	20 (5.2)	58 (8.2)
**Remission-phase specific**	66 (6.0)	37 (9.7)	29 (4.1)
**Diagnosis specific**	54 (5.0)	17 (4.5)	37 (5.2)
**End-of-life specific**	34 (3.1)	1 (0.3)	33 (4.7)
**Total**	**1092 (100)**	**382 (100)**	**710 (100)**

#### Main challenges and unmet needs identified

Across all posts, the main challenges included disease worsening (141 [12.9%]), psychological impact (112 [10.3%]), and the need to share experiences and seek support (94 [8.6%]). Table 5 describes the top eight challenges and unmet needs found in patient and caregiver posts, which constituted more than half of all occurrences identified in these posts (608 [55.7%]).

**Table 5:** Top eight challenges identified in posts from patients and caregivers

Challenge	Occurrence in all posts (*N* = 1092), *n* (%)	Occurrence in patient posts (*n* = 382), *n* (%)	Occurrence in caregiver posts (*n* = 710), *n* (%)	Category
**Progression/worsening/complication/recurrence of disease**	141 (12.9)	40 (10.5)	101 (14.2)	Disease specific
**Psychological impact**	112 (10.3)	38 (9.9)	74 (10.4)	Transversal
**Need for sharing/experiences/support**	94 (8.6)	42 (11.0)	52 (7.3)	Transversal
**Fear and management of aBC symptoms**	82 (7.5)	31 (8.1)	51 (7.2)	Disease specific
**Sequelae of aBC or care**	60 (5.5)	34 (8.9)	26 (3.7)	Remission-phase specific
**Worried about future and difficulties to project oneself**	46 (4.2)	14 (3.7)	32 (4.5)	Transversal
**Difficulty of delay in accessing treatment**	40 (3.7)	13 (3.4)	27 (3.8)	Treatment specific
**Consideration and management of pain**	33 (3.0)	11 (2.9)	22 (3.1)	Disease specific
**Total**	**608 (55.7)**	**223 (58.4)**	**385 (54.2)**	

aBC advanced bladder cancer

A saturation method was used to annotate challenges and unmet needs. Of 699 patient posts and 971 caregiver posts identified, 352 patient posts and 522 caregiver posts were annotated, after which no new categories of challenges or unmet needs were identified. Of the 352 patient posts and 522 caregiver posts analyzed, 202 and 323 posts, respectively, included at least one identified challenge or unmet need.

**Fig. 4:**
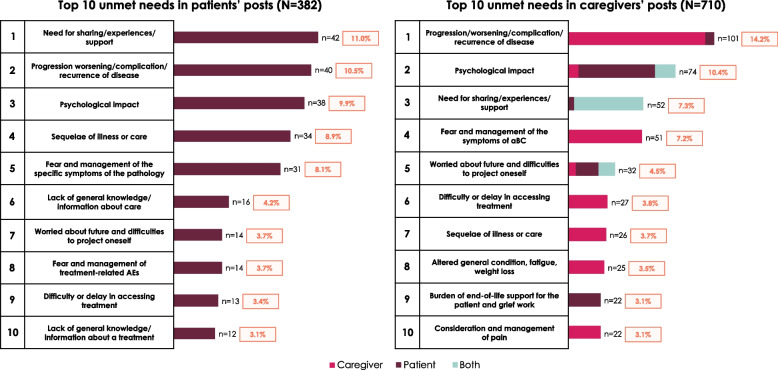
Main unmet needs and challenges mentioned in patients’ and caregivers’ posts. aBC, advanced bladder cancer; AE, adverse event

Across 382 patient posts and 710 caregiver posts that included identified instances of challenges and unmet needs, the need for experience sharing and support was mainly expressed in the form of questions from patients (42/382 [11.0%]) and their caregivers (52/710 [7.3%]). In addition, a lack of information and knowledge was expressed, with more specific questions about the management of aBC being asked by patients (16/382 [4.2%]) (Fig. 4) and their caregivers (15/710 [2.1%]) [34]. Questions specific to the treatments indicated for aBC were also found in some of the posts (12/382 [3.1%] for patients (Fig. 4) and 19/710 [2.7%] for their caregivers) [34].

In addition to challenges and unmet needs concerning patients, challenges concerning caregivers exclusively, or both patients and caregivers, were identified in caregivers’ posts. Among the main challenges described (Fig. 4), 3 challenges affected mostly caregivers in their posts about patients, i.e., psychological impact (74 [10.4%]), burden of caring for terminally ill patients and grieving (22 [3.1%]), and concerns about the future, with difficulties projecting ahead (32 [4.5%]).

### Patients

The need for sharing and support were the most common challenge in patients and found in 11.0% of posts (Fig. 4). Some patients actively shared their healthcare experiences on social media, seeking a supportive online “community” where others could relate to their journey. Patients commonly sought recommendations, opinions, and advice on various aspects of their healthcare, including diagnosis, treatment choices, and healthcare providers. Some, feeling isolated, reached out for reassurance and support from their digital peers, requesting encouragement, messages of hope, or prayers, as shown in the following message:

*“Good evening*,* I am 70 years old and have advanced bladder cancer. I am going to be operated in a few days and I would like to have testimonies of people living with an artificial bladder. Thank you very much*,* your help is precious to me” (France).*

Patients also expressed challenges related to the progression, worsening, or recurrence of their condition in 10.5% (40/382) of messages (Fig. 4). Due to the advanced or terminal nature of their medical condition, individuals who communicated their experiences online frequently divulged the intricate challenges that they encountered throughout the course of their illness. These challenges often encompassed the exacerbation of aBC, relapses, recurrences, and the emergence of new metastases. Patients tended to openly discuss these complications, not only to narrate their disease journey comprehensively, allowing other internet users to gain a deeper understanding of their circumstances, but also with the hope of finding solutions and assistance to address the difficulties arising from these complex issues. The following message described this situation:

*“Hello. My name is [NAME] is honestly I do not know where to start. In December 2017 I was diagnosed with bladder cancer*,* also in December I had my first surgery. At first checkup after 6 months (cystoscopy) everything normal. Second checkup December 2018 the tumor was back*,* I had surgery in January. Again checkup after 4 /5 month is …. the tumor is back again. I did pre-exams and waiting for the call for surgery we are in June 2019. My head keeps wandering*,* I don’t know what my future will be” (Italy).*

Challenges on the psychological level were also reported in 9.9% (38/382) of patients’ messages. Individuals with aBC endured persistent psychological distress throughout their medical journey. This included anxiety, fear, and stress experienced at various stages, from the initial diagnosis with feelings of powerlessness and uncertainty to moments of remission overshadowed by the fear of relapse. In addition to the demanding and prolonged nature of their care, patients also grappled with mental fatigue, which compounded their physical symptoms. This was described in the following message:

*“As written I am new here*,* am 66 years married and have 3 children*,* I was diagnosed with bladder cancer 4 months ago and the 1st operation is this week on Thursday*,* and should go again to the hospital to do the 2nd operation. I have to fight very much with it and at the moment I also have strong suicide thoughts*,* but then think of our 3 grandchildren who still need your grandpa. So that was it once*,* later maybe more” (Germany).*

Other major challenges or unmet needs include the sequelae of illness or care (8.9%), fear and management of the specific symptoms of the pathology (8.1%), and lack of general knowledge/scientific information about care (4.2%).

### Caregivers

Of 710 caregiver posts that included identified instances of challenges or unmet needs, psychological difficulties were the major challenges described in 10.4% (74/710) of caregivers’ messages (Fig. 4). Caregivers reported that caring for a loved one with aBC took a significant emotional toll on their mental health. They were intimately involved throughout the entire care process, which was emotionally taxing. This unanticipated and complex journey often left them feeling overwhelmed and unprepared. Providing physical, emotional, and practical support led to anxiety, depression, and exhaustion, which may persist even after their loved one’s passing, as described in the following posts:

*“Hello My mother was operated of a cancer of the bladder by cystectomy. Stage PTy 3*,* N0/25*,* R0. She had first undergone chemotherapy. What worries me is that it is written on the results of the scan that there were small hepatic hypodensites too small to be characterized. This makes me anxious. What do you think Doctor? Thank you” (France).*

*“Hello dear ones My mom got the results of her bladder cancer op today*, * not good the cancer is still there*,* now chemo since it has spread and see how it goes on…I’m just scared* * my dear*,* dear mom* * Why does this have to be now? Sorry had to puke many times….” (Germany).*

Caregivers also expressed challenges related to the end-of-life support for the patient as well as processing their own grief. The responsibilities placed on caregivers of individuals with aBC tended to intensify during the end-of-life and bereavement phases. As the patient’s condition deteriorated, caregivers often grappled with the overwhelming task of managing the patient’s symptoms, making them comfortable and dealing with end-of-life decisions, while coping with existing and anticipatory grief as they witnessed the disease progression and the decline in their loved one’s health. The grieving process following the patient’s passing can be exceptionally challenging for caregivers, as they may contend with feelings of isolation, hollowness, and guilt. To alleviate the weight of caregiving during this trying period, many caregivers turned to social media for support, sharing their experiences and seeking solace in the digital community. These feelings were described in the posts below:

*“They told me in January 2017 “maybe she lives till summer” (bladder tumor). She was with us for almost 3 more years and looked healthy. Unfortunately you are never ready for that moment. Hang in there ❤” (Italy).*

*“I’ve just lost my Dad x literally 6 weeks tomorrow and I still can’t believe he is gone. I can’t stop crying. As an only child I was so close to him and he was my hero. He didn’t even tell me he was ill. He died of Stage 4 bladder cancer. Collapsed at home on the Friday*,* dies on the Tuesday x I feel sad*,* angry*,* lost. He was my hero. What now????” (UK).*

Additionally, caregivers expressed their worries about the future and the challenge of projecting oneself. Caregivers supporting patients with aBC often faced substantial stress and the challenge of envisioning the future. They reported that the inherently unpredictable course of the disease and its uncertain prognosis posed significant hurdles for them in terms of predicting what lay ahead and being prepared to meet their loved one’s evolving needs. Despite their efforts to comprehend the intricacies of their loved one’s illness, including treatment options and forthcoming surgeries, a shroud of ambiguity persisted, making anticipation a difficult task. Furthermore, the prevailing fear of losing their cherished family member loomed large in the minds of caregivers, casting a pervasive shadow over every aspect of caring for the patient. These emotions were illustrated in the following messages:

*“I thank you*,* but unfortunately she has bladder cancer*,* the only hope is that it will not increase through medication and that what she has left to live will be without suffering” (Italy).*

*“My dad has terminal bladder cancer. He’s detoriated rapidly this week. He isn’t getting out of bed*,* stopped eating and has now stopped drinking. He’s constantly sleeping How long can he live like this?” (UK).*

The reality of being a caregiver was also reported (1.8%), given the demanding and time-consuming nature of their responsibilities. This included the financial strain of caregiving, which could be particularly arduous and often necessitating caregivers to make sacrifices, such as reducing their paid work hours or leaving their jobs altogether to attend to their ailing family member. Additionally, caregivers reported feelings of social isolation, a consequence of their caregiving duties that frequently curtailed their ability to engage in social activities and nurture their relationships.

## Discussion

The objective of the study was to understand patient and caregiver perceptions and to identify gaps and unmet needs to help improve the management of aBC and to better address patients’ needs and expectations.

Our study revealed a discernible impact from aBC on QoL, wherein 40.0% (558/1396) of users mentioned at least an impact on one aspect of QoL. Among those users’ messages, a negative impact on physical well-being was consistently experienced across the Eu5, with 56.8% (317/558) of patients and caregivers encountering physical challenges. Furthermore, psychological well-being emerged as another dimension substantially influenced, with 48.6% (271/558) of users facing psychological challenges. Most unmet needs and challenges identified belonged to two main categories: transversal (307/1092 [28.1%]) and disease specific (295/1092 [27.0%]). Remarkably, the top three unmet needs mirrored each other for patients and caregivers alike. These included the need for sharing and support (42/382 [11.0%] for patients and 52/710 [7.3%] for caregivers), challenges related to the progression or worsening of their condition (40/382 [10.5%] for patients and 101/710 [14.2%] for caregivers), and issues with their psychological well-being (38/382 [9.9%]) for patients and 74/710 [10.4%] for caregivers).

The findings on the negative physical and psychological impacts on QoL align with findings from previous research in BC. Notably, patients diagnosed with non–muscle-invasive BC and the more aggressive muscle-invasive BC reported substantial declines in their health-related QoL scores over time, whether compared to individuals without a history of cancer [35] or before and after the cancer diagnosis [36]. The adverse impact on physical, mental, and social aspects of QoL was more pronounced among patients with muscle-invasive BC [35, 37]. In interviews regarding locally advanced or metastatic urothelial carcinoma, patients, caregivers and physicians reported pain as the main symptom after diagnosis. Depression and sadness were also commonly reported [38].

Our analyses extended beyond the patient perspective to encompass the impact on caregivers; a phenomenon that aligns with other research is that as the disease progressed, it increasingly impacted caregivers’ QoL [39]. This is particularly accentuated in the context of palliative care and end-of-life support for cancer patients, underscoring the pivotal role of caregivers, albeit at the cost of their own mental well-being [40]. Furthermore, other research aligned with our findings, showing that caregivers primarily reported significant emotional challenges, such as fear, worry, sadness, physical issues, and fatigue [41]. Decreased well-being was particularly noted among female caregivers or those caring for patients with aBC. Caregivers found substantial support in web-based information and personal stories from other caregivers [41]. This research highlights the need for enhanced patient and caregiver support and improvements in the overall care pathway, which could be facilitated by raising disease awareness and providing more comprehensive training for healthcare professionals [42]. Our study highlighted the unmet needs related to disease progression and psychological impacts. This observation is congruent with earlier research, where worsening of the patients' physical condition rendered routine activities, such as bathing and toileting, increasingly challenging [9, 43]. The physical limitations incurred and the taxing treatments and procedures contributed to heightened levels of anxiety, depression, and distress [44, 45]. A prominent theme that emerged was the need for support, as articulated by both patients and caregivers. Many turned to social media for support and information, revealing a lack of information regarding treatment options and symptom management [9, 46, 47]. This highlights the significance of emotional support and the pervasive information gap encountered by patients with aBC and their caregivers [48–50].

Despite established clinical guidelines [51, 52] and the availability of effective systemic anticancer treatments [14, 15, 53], the majority of patients with aBC go untreated [54] due to complex factors, including health system aspects of the care pathway in aBC diagnosis and therapy, and patient and caregiver factors. Addressing these concerns can encourage timely treatment seeking, further contributing to improving treatment rates [55].

It is important to recognize the demographic profile of the patients involved in our study. Our study population was primarily comprised of older males, a group traditionally characterized by limited engagement with social media platforms. A noteworthy observation is that family members, most frequently a daughter, actively engaged on social media on their behalf, thus ensuring that we were able to capture insights from this demographic.

Social media platforms are increasingly being used by internet users not only to generate, access, and share health-related information and experiences, but also to express emotions and sentiments that are unsolicited and unfiltered [18–20]. This makes social media listening an effective tool for exploring the perceptions of patients and caregivers, offering comprehensive context to understand patients’ experiences with conditions such as aBC. The 2020 guidelines from the US Food and Drug Administration recognize social media as an appropriate method for gathering patient input, citing its utility in accessing difficult-to-reach populations and its ability to provide accurate and automatic capture of data, and possibly result in greater self-disclosure by patients [56]. This approach not only facilitates the collection of a wide array of patient experiences but also ensures the inclusion of voices that might otherwise be overlooked. Furthermore, the International Society for Pharmacoeconomics and Outcomes Research highlights that data collected from social media sources can spotlight issues most relevant to patients and their families, emphasizing the value of these insights in complementing traditional research methods [57]. By evaluating patients and their caregivers’ perspectives in their own words, without any input from healthcare professionals, our study was able to gather fresh and unbiased insights. Despite ethical challenges associated with consent, the data for our study were collected from publicly accessible sources without personal identifiers, ensuring respect for privacy and confidentiality.

Our findings highlight the substantial impact of physical and psychological challenges on patients with aBC and their caregivers in the Eu5. These insights emphasize the need for comprehensive support, education, and community building for patients with aBC and their families. Creating patient and caregiver communities, both online and offline, can facilitate emotional support. Comprehensive care pathways may ensure coordinated physical and psychological support. Healthcare providers and policymakers can collaborate to address the physical and psychological challenges, bridge gaps in aBC management, and enhance the overall well-being of those affected by aBC. The results of this study may assist patients, caregivers of people with aBC, and clinicians in shared decision-making and ensure patients receive the type of aBC-specific care that most aligns with their personal treatment priorities. For clinicians, familiarization with the terms and language used by patients and their caregivers on social media platforms may help guide discussions about symptoms, burdens, and treatment options.

### Limitations

This study shares the inherent limitations of social media research, namely that patient-provided information is both authentic and voluntarily shared in the public domain. This analysis relied solely on publicly available social media posts, precluding data collection from non-public platforms such as closed Facebook groups. The quality of insights derived from digital conversations relied upon the depth of information disclosed by patients and caregivers encompassing details concerning their health condition, unmet needs, disease management challenges, and overall QoL. In cases where information was lacking, context and representativeness became uncertain. The small sample size, primarily influenced by the demographic makeup of patients with aBC and elderly caregivers, limited the depth of analysis. Data quality also depended on the digital literacy of users, affecting their ability to convey information accurately, including specific details such as BC type, stage, and treatment. These self-reported data may be susceptible to recall bias, relying on memory and personal recollection. Individuals who contribute to social media discussions may not accurately represent the broader patient population encompassing aBC and their caregivers. We are unable to verify contributor characteristics, such as demographics and clinical information, which could limit the accuracy of testimonials. Moreover, it is worth noting that relevant posts might have been mistakenly eliminated during the filtering process, and the potential for duplication exists in cases where users were engaged on multiple forums. To reduce background noise, we used threshold values similar to those in our previous work [58–60], which may have constrained the natural processing analysis. Lastly, we applied the saturation method to random portions of the dataset instead of the entire set, raising the possibility of missing some information related to patients’ and caregivers’ unmet needs. Despite these limitations, this study provides valuable insights into the unmet needs of both patients with aBC and their caregivers derived directly from their input.

## Conclusions

To our knowledge, this study represents the first retrospective analysis of publicly available social media data on the experiences of patients with aBC and their caregivers within the Eu5. It is apparent that aBC, a prevalent and often life-threatening cancer that exerts a large economic burden on healthcare systems, also inflicts profound emotional and physical distress on affected individuals. These insights can offer physicians and multidisciplinary care teams a deeper understanding of the challenges faced by patients with aBC and their caregivers. It highlights the opportunity to enhance physician-patient communication and to institute earlier and better support systems to address these issues effectively. In parallel, access to effective and well-tolerated systemic anticancer treatments is crucial to enhance patient outcomes and improve the overall experience for both patients and their caregivers. Future research, encompassing both qualitative and quantitative approaches, should validate and build on these findings, thereby advancing our knowledge of the impact of aBC on patients and their families.

## Supplementary Information

Supplementary Material 1.

## Data Availability

The algorithms used are proprietary intellectual property of Kap Code and, as such, cannot be disclosed. Additionally, owing to compliance with the General Data Protection Regulation (Regulation [EU] 2016/679), these data cannot be shared.

## Abbreviations

aBC: Advanced bladder cancer
AE: Adverse event
BC: Bladder cancer
EU: European Union
Eu5: UK, France, Italy, Spain, and Germany
QoL: Quality of life
UK: United Kingdom
