# Supplementary material for: Perceived unmet needs and impact on quality of life of patients living with advanced bladder cancer and their caregivers: results of a social media listening study conducted in five European countries
Source: BMC Cancer. 2024 Nov 25;24:1444. doi: 10.1186/s12885-024-13092-x (PMC11587617; doi:10.1186/s12885-024-13092-x)
Supplement: Supplementary file 1 — Supplementary Material 1. [file 12885_2024_13092_MOESM1_ESM.docx]

**Supplementary Information/Appendix 1**

**Query used for extraction on Brandwatch:**

**France: (((vessie OR ureter OR urothelial OR urotelial OR uro-telial OR "uro telial" OR uro-thelial OR "uro thelial" OR transitionnel) NEAR/5 (Cancer OR tumeur OR carcinome OR Adenocarcinome OR Sarcome))**

**UK: ((("transitional cell carcinoma" OR "transitional urothelial cell carcinoma" OR "urothelial cell carcinoma" OR ((Bladder OR Urothelial OR Urotelial OR "BC") NEAR/5 (Cancer OR carcinoma OR tumor OR sarcom))) NOT ("BC" AND "Breast"))**

**Germany: (Blasenkrebs OR Blasen-krebs OR Harnblasenkrebs OR Harnblasen-krebs OR Blasentumor OR Blasen-tumor OR Blasenkarzinom OR Blasen-karzinom OR Harnblasenkarzinom OR Harnblasen-karzinom OR Urothelkarzinom OR Urothel-Karzinom OR  Übergangszellkarzinom OR  Transitionalzellkarzinom OR Übergangszell-karzinom OR Transitionalzell-karzinom OR ((Blase OR Harnleiter OR Harnweg OR Harnblase OR Nierenbecken OR Harnröhre OR Nieren OR Urothel) NEAR/6 (Krebs OR Tumor OR Karzinom OR Adenokarzinom OR Scheiß OR Transitionalzell OR Übergangszell)))**

**Italy: (((Vescica OR "Sacca piriforme" OR Serbatoio OR Uretere OR Ureteri OR Uroteliale OR Urotelioma OR transizionale OR transizionali OR transizione OR vescicali OR vescicale) NEAR/6 (Cancro OR Tumore OR Carcinoma OR Adenocarcinoma OR Sarcoma OR tumorali OR Neoplasia))**

**Spain: ((Vejiga OR Vegiga OR" Bejiga OR Begiga OR Ur?ter OR Urotelio OR Urotelial OR Uro-telial OR Uro telial OR Uretra OR C?lulas de transici?n OR Epitelio de transici?n" OR "C?lulas escamosas" OR "C?lulas peque?as" OR Vesical) NEAR/6 (C?ncer OR C?nser OR Tumor OR Bulto OR Quiste OR Carcinoma OR Adenocarcinoma OR Sarcoma OR Masa OR Foco OR Bola OR Pelota OR Polipo))**

**Keywords used to select posts regarding aBC**

| Dataset | Keywords |
| --- | --- |
| France | **(avancé OR general OR metasta OR terminal OR stade3 OR stade4 OR stadeIII OR stadeIV OR grade3 OR grade4 OR gradeIII OR gradeIV OR ((stade OR grade OR niveau) NEAR/6 ("3" OR "3a" OR "3b" OR "4" OR "4a" OR "4b" OR "III" OR "IIIa" OR "IIIb" OR "IV" OR "IVa" OR "IVb" OR "T3" OR "T=3" OR "T3a" OR "T=3a" OR "T3b" OR "T=3b" OR "T4" OR "T=4" OR "T4a" OR "T=4a" OR "T4b" OR "T=4b" OR "M1" OR "G3" OR "G3a" OR "G3b" OR "G4" OR "G4a" OR "G4b" OR "GIII" OR "GIIIa" OR "GIIIb" OR "GIV" OR "GIVa" OR "GIVb")) OR invasif OR invasive OR infiltrant OR infiltre OR ((ganglion OR Cancer OR tumeur OR carcinome OR Adenocarcinome OR Sarcome OR Kancer OR "K" OR "kc" OR tumoral OR crabe) NEAR/15 (touche OR etendu OR sorti OR propag OR envahi OR progress)) OR "TVIM"))** |
| United Kingdom | (advanced OR "locally advanced" OR metasta OR metastici OR "mets" OR terminal OR "stage 3" OR "stage III" OR "stage IV" OR "stage 4" OR "stage IIIa" OR "stage 3a" OR "stage 3b" OR "stage IIIb" OR "stage IVa" OR "stage 4a" OR "stage IVb" OR "stage 4b" OR "muscle invasive non-metastatic" OR "muscle-invasive non-metastatic" OR "muscle invasive non metastatic" OR "muscle invasive metastatic" OR "muscle invasive" OR "muscle-invasive" OR infiltrat OR "MIBC" OR "M1" OR "M=1" OR "N1" OR "N=1" OR "N2" OR "N=2" OR "N = 2" OR "N3" OR "N=3" OR "invasive bladder cancer" OR (("lymph node" OR "lymph nodes") NEAR/5 ("spread" OR "touched" OR "cancer in" OR "present in" OR "found in" OR propag)) OR (("T3" OR "T=3" OR "T3a" OR "T=3a" OR "T3b" OR "T=3b" OR "T4" OR "T=4" OR "T4a" OR "T=4a" OR "T4b" OR "T=4b") NEAR/5 (stag OR grad))))  NOT ("non muscle" OR "non-muscle" OR "NMIBC") |
| Germany | (fortgeschritten OR gestreut OR ausgebreitet OR Metastase OR metastasiert OR Endstadium OR ((Stadium OR Stufe OR Grad OR Grading OR Krebs OR Tumor OR Karzinom OR Adenokarzinom OR Scheiß OR Transitionalzell) NEAR/6 ("3" OR "3a" OR "3b" OR "4" OR "4a" OR "4b" OR "III" OR "IIIa" OR "IIIb" OR "IVa" OR "IVb" OR "T3" OR "T3a" OR "T3b" OR "T4" OR "T4a" OR "T4b" OR "T=3" OR "T=3a" OR "T=3b" OR "T=4" OR "T=4a" OR "T=4b" OR alto OR terzo OR "G3" OR "G4" OR "G5" OR "G 3" OR "G 4" OR "pT3" OR "pT4" OR highgrade OR high)) OR invasiv OR infiltrativ OR "Metastasiertes Blasenkarzinom" OR Lymphknotenmetastase OR Lymphdrüsenkrebs OR Lymphbefall OR Lymphknotenbefall OR  ((Lymph OR Lymphsystem OR Lymphknoten OR Lypf OR Lypfknoten OR Lümpf OR Lymf OR Lümf OR Krebs OR Tumor OR Karzinom OR Adenokarzinom OR Scheiß OR Transitionalzell) NEAR/5 (befall OR erreich OR entnomm OR entfern OR ausgeweit OR ausgebreit OR übergeg)) OR "UKMP") |
| Italy | (Avanzato OR "Localmente Avanzato" OR Metastatizzato OR Metastatico OR "In metastasi" OR "Terminale" OR ((Stadio OR Grado OR Livello OR Stadiazione OR Cancro OR Tumore OR Carcinoma OR Adenocarcinoma OR Sarcoma OR tumorali OR Neoplasia) NEAR/6 ("3" OR "3a" OR "3b" OR "4" OR "4a" OR "4b" OR "III" OR "IIIa" OR "IIIb" OR "IVa" OR "IVb" OR "T3" OR "T3a" OR "T3b" OR "T4" OR "T4a" OR "T4b" OR "T=3" OR "T=3a" OR "T=3b" OR "T=4" OR "T=4a" OR "T=4b" OR alto OR terzo OR "G3" OR "G4" OR "G5")) OR invasivo OR invasiva OR infiltrante OR "muscolo-infiltrante" OR "muscolo infiltrante" OR ((Ghiandola OR Ghiandole) NEAR/5 (colpito OR colpite OR esteso OR propagato OR invaso)) OR "MIBC")) |
| Spain | ((Bufeta OR "Bufeta urin?ria" OR Ur?ter OR Vesical OR Uroteli OR Urotelial OR Uro-telial OR "C?l·lules urotelials" OR "C?l·lulas urotelials" OR "C?lules urotelials" OR "C?lulas urotelials" OR "C?l·lules de transici?" OR "C?lules de transici?" OR "C?lulas de transici?" OR "C?l·lulas de transici?" OR "Epiteli de transici?" OR "C?l·lules escamoses" OR "C?lules escamoses" OR "C?l·lulas escamoses" OR "C?lulas escamoses" OR "C?l·lules petites" OR "C?lules petites" OR "C?l·lulas petites" OR "C?lulas petites") (C?ncer OR Tumor OR Bony OR Bulto OR Bult OR Bultu OR Quist OR Quiste OR Adenocarcinoma OR Sarcoma OR Focus)) |
